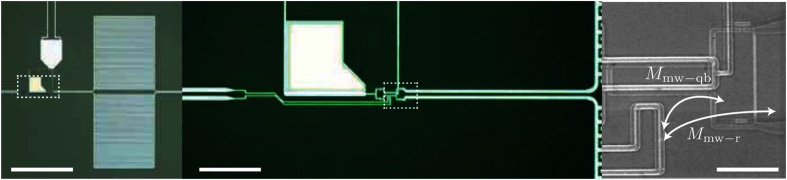# Corrigendum: Broken selection rule in the quantum Rabi model

**DOI:** 10.1038/srep35093

**Published:** 2016-10-19

**Authors:** P. Forn-Díaz, G. Romero, C. J. P. M. Harmans, E. Solano, J. E. Mooij

Scientific Reports 6: Article number: 2672010.1038/srep26720; published online: 06 07 2016; updated: 10 19 2016

In this Article, there is an error in Figure 6. The arrows in the right-hand portion of the image are misplaced. The correct Figure 6 appears below as Figure 1.

## Figures and Tables

**Figure 1:**